# Comparative analysis of the composition of intestinal bacterial communities in *Dastarcus helophoroides* fed different diets

**DOI:** 10.1093/jis/14.1.111

**Published:** 2014-08-12

**Authors:** Wei-Wei Wang, Cai He, Jun Cui, Hai-Dong Wang, Meng-Lou Li

**Affiliations:** 1 Laboratory of Forestry Pests Biological Control, College of Forestry, Northwest A&F University, Yangling, Shaanxi, 712100, P. R. China; 2 Wuwei Academy of Forestry Sciences, Wuwei, Gansu, 733000, P. R. China

**Keywords:** parasitoid beetle, nested PCR, DGGE, larva, adult

## Abstract

The diversity of the intestinal bacterial communities in *Dastarcus helophoroides* (Fairmaire) (Coleoptera: Bothrideridae) larvae and adults was assayed by PCR-DGGE to determine whether different artificial diets could influence these bacterial communities. Two diets were used for feeding the larvae and four for the adults. *Escherichia, Desemzia, Staphylococcus, Asticcacaulis, Cellvibrio, Aurantimonas,* and *Planomicrobium* were isolated from the gut of the adults, with *Escherichia* and *Staphylococcus* being the main bacterial communities, and the quantities of intestinal bacterial were different in the adults fed different diets. Specifically, the amount of intestinal bacteria from the adults fed different diets had the following ranking according to the major component of the diet: ant powder > darkling beetle pupa powder > cricket powder > silkworm pupa powder. *Escherichia, Bacillus, Staphylococcus, Kurthia, Planococcaceae, Ralstonia, Leptothrix, Acinetobacter,* and *Pseudomonas* were isolated from the gut of the larvae. The quantity of intestinal bacteria from the larvae fed the darkling beetle pupae was greater than that from the larvae fed other artificial diets. This study, for the first time, investigated the effect of artificial diets on the bacterial community and the intestinal microbial diversity of *D. helophoroides* .

## Introduction

Vast numbers of microorganisms inhabit the insect gut, and many occupy the alimentary tract and play an important role in the physiological function and behavior of their host ( Dillon and Dillon 2004 ). It is well known that intestinal tract flora plays an important role in maintaining the health of the host by maintaining a normal ecological equilibrium, combining absorption, digestion, and protection against the invasion of pathogenic microorganisms ( Rio et al. 2004 , Dillon et al. 2005 , Jeon et al. 2011 ). At present, the research on the insect intestinal microflora and dominant bacterial communities is focused mainly on termites belonging to Isoptera and on *Bombyx mori* (L.), which belongs to Lepidoptera ( Eutick et al. 1978 , Brune and Friedrich 2000 , Xiang et al. 2007 ).

Denaturing gradient gel electrophoresis (DGGE) has been used widely to identify and monitor the microbes common to different individuals and environments ( Reeson et al. 2003 , Hayashi et al. 2007 ). Using polymerase chain reaction and denaturing gradient gel electrophoresis (PCR-DGGE), Zhang and Jackson (2008) studied the digestive tract of scarabs and found that the dominant bacterial communities were mainly Clostridiales, followed by Proteobacteria and Bacteroides. Twenty-one bacteria, belonging to thirteen microflora, were isolated from the larval gut of *Apriona germari* (Hope) ( Zhang et al. 2004 ). Different food resources provide nutrients to the different bacterial communities in the intestines of insects, and the diet of a number of aquatic invertebrates is known to influence their intestinal microflora ( Harris 1993 ). The effect of diet on the intestinal bacteria in crickets ( Kaufman et al. 2000 ), cockroaches ( Kane and Breznak 1991 ), and wasps ( Reeson et al. 2003 ) has been reported.

Kane and Breznak (1991) and Kaufman et al. (2000) found that using different food sources could promote the development of alternative bacterial communities in cockroaches and crickets. Abundant microorganisms inhabit in the gut of *Costelytra zealandica* (White) of Coleoptera, an important group in insects ( Zhang and Jackson 2008 ), and are considered to play important roles in coleopteran nutrition and metabolism ( Jeon et al. 2011 ).

*Dastarcus helophoroides* (Fairmaire) (Coleoptera: Bothrideridae) is one of the most effective natural enemies against the large-bodied longhorn beetle ( Yang 2004 ). The larva of *D. helophoroides* is an ectoparasitoid of the larvae and pupae of various trunk borers, such as *Anoplophora glabripennis* (Motschulsky), *Monochamus alternatus* (Hope), *Mallambyx radei* Blessig, *Apriona swainsoni* (Hope), *Batocera horsfieldi* (Hope), and *A. germari* ( Wang et al. 1996 , Yang 2004 ). Although the adult lifespan of *D. helophoroides* exceeds three years, the larval stage is only approximately one week, and the pupal stage is 20 to 30 days. The previous reports on *D. helophoroides* were focused mainly on its biological characteristics ( Lei et al. 2003 ), artificial diets, the release of adults for biological control ( Wang and Ogura 1999 ), and the adult host-identification mechanism of using informational chemicals ( Wei et al. 2008 ). In this study, we examined the microbial communities of larval and adult guts of *D. helophoroides* by using DGGE to separate amplified *16S rDNA* gene fragments by PCR.

Because the narrow distribution and low density of *D. helophoroides* in nature limit its function in biological control, it is necessary to develop artificial diets for *D. helophoroides.* However, if the composition of the artificial diet were inappropriate, it would affect the intestinal bacterial community and thus the digestive function and healthy growth of the insect. To date, artificial diets for *D. helophoroides* larvae and adults have been investigated ( Wang et al. 1999 , Lei et al. 2005 , Shang et al. 2009 ), but whether these diets have an effect on the intestinal bacteria remains unknown. Hence, this study investigated the larval and adult intestinal microflora of *D. helophoroides* fed different artificial diets under the same conditions.

## Materials and Methods

### Diets and insects

All of the insects used in this study were housed in the laboratory under optimal growing conditions. The adult artificial diets (Diet I, II, III, and IV) were prepared as shown in Table 1 , and all insects used as the major component of four diets were collected from the wild, dried, and ground into powder in the lab. The larval Diet V consisted of darkling beetle pupae. The artificial Diet VI was prepared as shown in Table 2 , and the contents were mixed well and stored at 5°C. The larvae and adults of *D. helophoroides* used in the experiment were maintained in the Forestry Ento mology Laboratory at Forestry College, North west Agriculture and Forestry University, China. The larval feeding method was according to Shang et al. (2009) , and sixth-instar larvae were used in the experiments. The adult feeding method was performed according to the procedure described by Lei et al. (2005) , and the experimental feeding period was from 22 August 2011 to 3 November 2011, 72 days to ensure the formation of intestinal microflora. All of the larvae and adults were maintained and fed in controlled incubators at 25 ± 1°C and 60-70% RH with a photoperiod of 16:8 L:D.

**Table 1.**
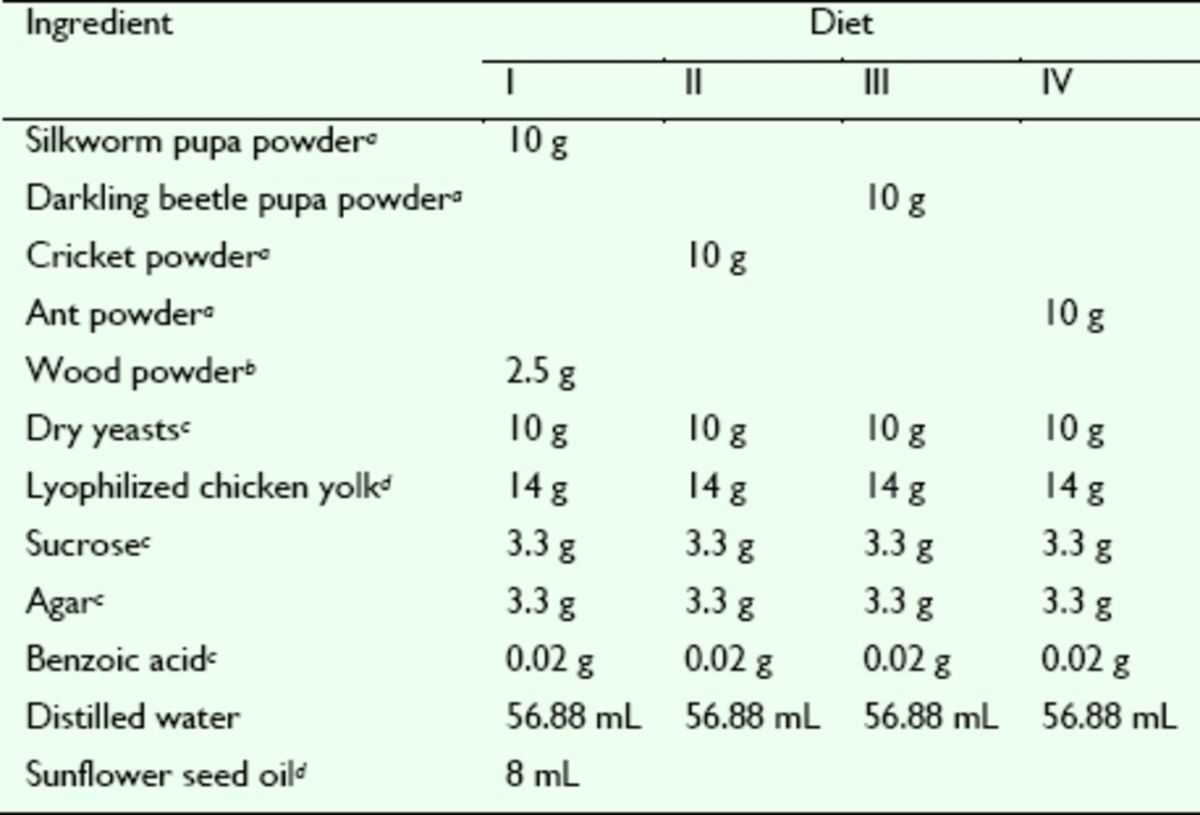
Composition of the four diets used for feeding *D. helophoroides* adults.

^*a*^ Silkworm pupae, crickets, darkling beetle pupae, and ants were collected from the wild and then dried and ground into powder in the lab.

^*b*^ Poplar xylem was dried and ground into powder in the lab.

^*c*^ Dry yeasts, sucrose, agar, and benzoic acid were purchased from TIANGEN (Beijing, China).

^*d*^ Lyophilized chicken yolk and sunflower seed oil were purchased from the supermarket.

**Table 2.**
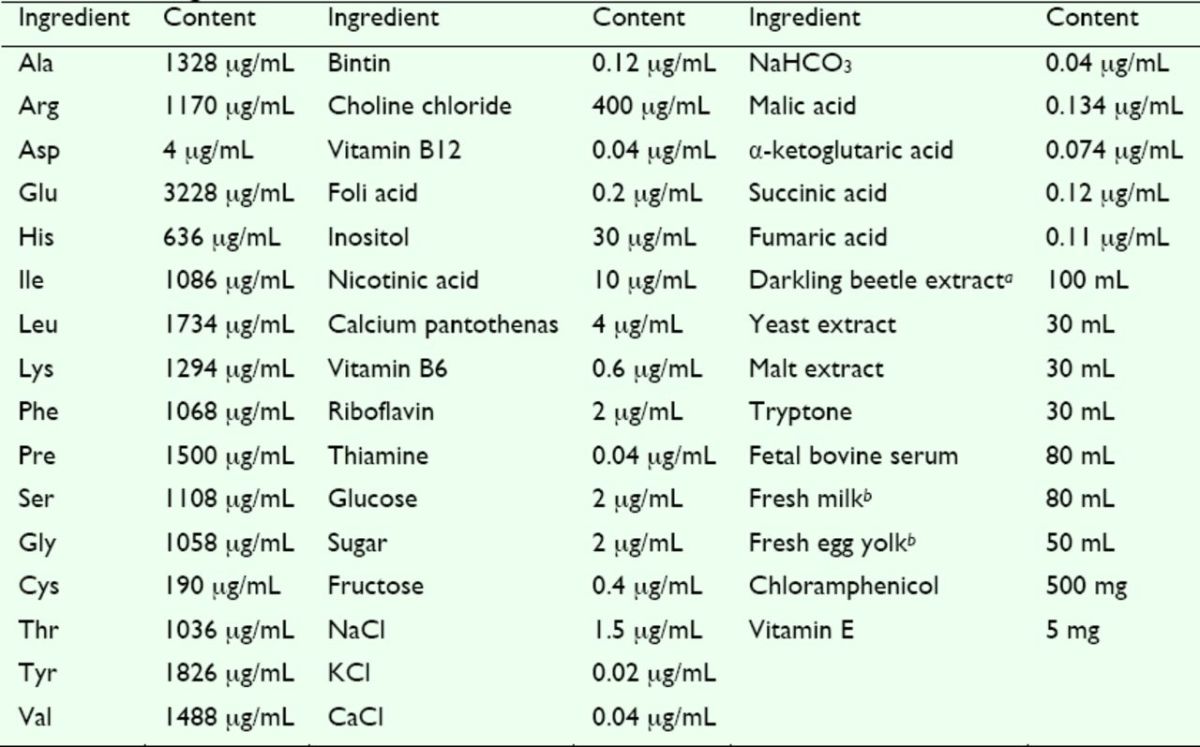
The ingredients and contents of artificial Diet VI

All ingredients except those indicated were purchased from TIANGEN (Beijing, China).

^*a*^ Darkling beetle pupae were used to produce darkling beetle extract in a juicer (JYZ-B550, China).

^*b*^ Fresh milk and fresh egg yolk were purchased from the supermarket.

### DNA extraction

Samples of 10 individuals were collected randomly from each diet and dissected to remove the guts, which were pooled, gently homogenized using a pestle, and mixed with STE buffer (0.1 M NaCl, 0.01 mM Tris-HCl [pH 8.0], and 0.001 M EDTA [pH 8.0]). The supernatant was incubated with proteinase K (200 ug/mL) and 1% (w/v) sodium dodecyl sulfate (SDS) at 55°C for 5 hr and then centrifuged at 12,000 × *g* for 10 min at 4°C. The clear supernatant was extracted once with an equal volume of TE buffer-saturated phenol-chloroform-isoamyl alcohol (25: 24: 1) and once with equal volume of TE buffer-saturated chloroform-isoamyl alcohol (24: 1). The DNA was precipitated with two volumes of 100% ethanol at -20°C for 20 min, pelleted by centrifugation at 12,000 × *g* for 10 min, washed twice with 70% ethanol, and resuspended in 30-50 μL 1 × TE, according to standard techniques ( Hughes et al. 1993 ).

### 16S rDNA nested PCR amplification

Because the gut microbial DNA is a mixture, nested PCR ( Soejima et al. 2011 ) was used to amplify the bacterial full-length *16S rDNA* gene first and then to amplify the bacterial 16S rDNA V3 region. The universal primers 27 mf (5'- AGA GTT TGA TCM TGG CTC AG -3') and 1492r (5'- ATG GGY TAC CTT GTT ACG ACT T -3') ( Weisburg et al. 1991 ) were used to amplify the full-length *16S rDNA* gene. The amplification reactions were performed in a 25 µL final volume containing 12.5 µL 2 × *ES* master, 1 µL of each primer (10 µmol/L) and 1 µL genomic DNA (1 ug/mL). The cycling protocol included the following: an initial denaturation at 95°C for 5 min; 29 cycles of denaturation at 94°C for 60 sec, annealing at 55°C for 30 sec, and extension at 72°C for 90 sec; and a final extension step at 72°C for 10 min. The PCR products were diluted 30 times in RNA-free water and used to amplify the V3 region.

The bacteria-specific primers 341f (5'- CCT ACG GGA GGC AGC AG -3') and 534r (5'-ATT ACC GCG GCT GCT GG -3') and the forward primer with a GC-clamp at its 5 **'** end ( Muyzer et al. 1993 ) were used to amplify the V3 variable region of the *16S rDNA* gene. The PCR mixture (25 µL) contained 12.5 µL 2 × *ES* master, 1 µL of each primer (10 µmol/L) and 1 µL of the first PCR product diluted 30 times. The touchdown PCR cycling protocol included the following: an initial denaturation at 94°C for 5 min; 18 touchdown cycles of denaturation at 94°C for 45 sec, annealing at 65°C (-0.8°C per cycle) for 45 sec, and extension at 72°C for 45 sec, followed by 7 cycles of 94°C for 45 sec, 55°C for 45 sec, and 72°C for 45 sec; and a final extension step of 72°C for 10 min to eliminate artifactual double bands ( Janse et al. 2004 ). The products of the touchdown PCR were electrophoresed through a 1.5% (w/v) TAE agarose gel containing ethidium bromide to ascertain the size and amount of the products.

### Denaturing gradient gel electrophoresis

Denaturing gradient gel electrophoresis (DGGE) of the amplified *16S rDNA* gene was performed using an 8% acrylamide gel containing a denaturant gradient of 40-60% (100% defined as 7 M urea and 40% deionized formamide) in 1 × TAE buffer (40 mM Tris, 20 mM acetate, and 1.0 mM Na2-EDTA) at 60°C at a constant voltage of 80 V for 1 hr and 60 V for 16 hr. The gels were stained with ethidium bromide and photographed with UV transillumination. The DNA fragments from the DGGE gels were excised with sterile razor blades immediately after staining and visualization and soaked in sterilized distilled water at 4°C overnight. The supernatant after centrifugation (10,000 × *g* , 5 min at 4°C) was used as the DNA template for the 16S rDNA V3 amplification using the same primers without the GC-clamp. The PCR products were analyzed by electrophoresis on a 1.5% (w/v) agarose gel. The amplification products were purified using a universal DNA purification kit (TIANGEN, www.tiangen.com ) and sequenced.

### DGGE image processing and data analysis

The bacterial *16S rDNA* gene sequences were subjected to an NCBI nucleotide blast search ( http://blast.ncbi.nlm.nih.gov/Blast.cgi ) to identify the sequences of the highest similarity. Images of the DGGE gels were digitized and analyzed using Quantity One software (version 4.6.2, Bio-Rad, www.bio-rad.com ). The bands were identified by examining the magnified images, absorption peaks, and similarity in each lane.

Magurran (1988) used the Shannon index ( *H* ) to evaluate the biodiversity of both soils and enrichment culture. The Shannon index of intestinal bacteria was calculated based on the number and intensity of bands present on DGGE samples, run on the same gel, according to equation [1]:

$H = -\sum P_i logP_i$

[1]

where *P*_i_ is the important probability of the bands in a gel lane.

*P*_i_ was calculated with equation [2]:

$P_i = n_i/N$

[2]

where *n _i_* is the band intensity for each individual band and *N* is the sum of intensities of bands in a lane.

Gel analysis included conversion of the scanned gel image and normalization in order to correct shift within or between gels, so that bands or peaks of the same molecular size had the same physical position relative to a standard. Once all banding profiles were in a standardized analysis format, each band could be described by its position on the gel and by its relative intensity ( Andreoni et al. 2004 ).

## Results

### Nested PCR amplification of henomic DNA

**Fig. 1.**
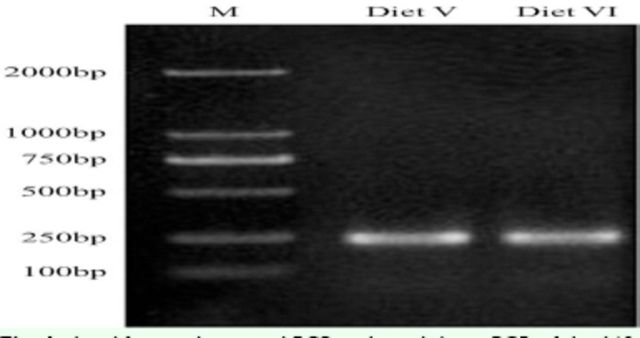
Amplification by nested PCR and touchdown PCR of the 16S rDNA V3 regions of larval intestinal bacteria of *D. helophoroides* fed different diets; M, DNA marker. The essential component of Diet V was powdered darkling beetle pupae; the essential components of Diet VI are listed in Table 2 . The sizes of PCR products were approximately 200 bp, and no non-specific amplification was noted.

**Fig. 2.**
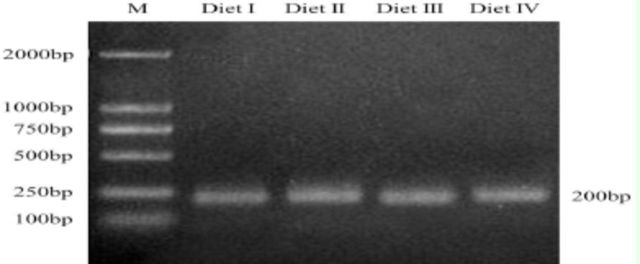
Amplification by nested PCR a nd touchdown PCR of the 16S rDNA V3 regions of adult intestinal bacteria of *D. helophoroides* fed different diets; M, DNA marker. The essential components of each diet are listed in Table 1 . The sizes of PCR products were approximately 200 bp, and no non-specific am plification was noted.

Nested PCR amplifications of the 16S rDNA V3 regions were performed using genomic DNA samples isolated from the guts of *D. helophoroides* larvae and adults fed different diets. The sizes of the nested PCR products were approximately 200 bp ( Figs. 1 and 2 ), and no non-specific amplification was noted.

### DGGE profiles of intestinal bacteria

**Fig. 3.**
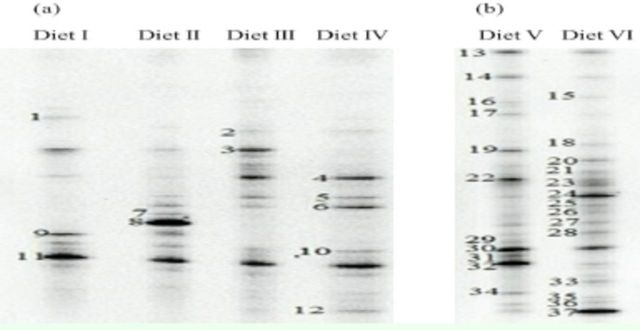
DGGE analysis of the bacterial *16S rDNA* genes in the guts of (a) adults and (b) larvae of *D. helophoroides* fed different diets. Diets I–IV were the adult diets, Diets V a nd VI were the larval diets. The numbers indicate sequenced bands; see Table 4 .

**Fig. 4.**
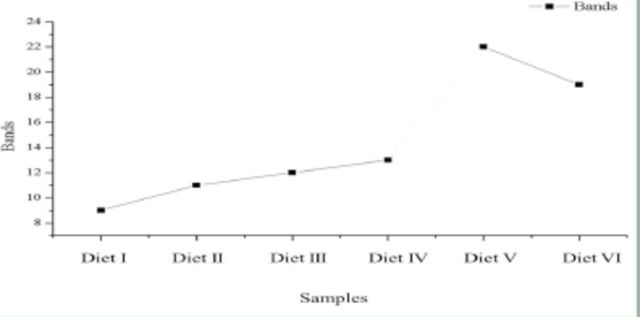
The bands of the denaturing gradient gel electrophoresis (DGGE) profiles of the intestinal bacteria of *D. helophoroides* larvae and adults fed different diets. Diets I–IV were the adult diets, Diets V and VI were the larval diets.

The predominant DGGE bands and quantitative changes in the intestinal bacteria of *D. helophoroides* fed different diets are shown in Fig. 3 and Fig. 4 , respectively. The quantitative order (number of bands detected in the intestines) of the different diets fed to the adults was Diet IV > Diet III > Diet II > Diet I, and the order for the larval diets was Diet V > Diet VI ( Fig. 4 ).

Table 3 illustrates the diversity of intestinal bacterial communities of the larvae and adults fed different diets. Each DGGE profile was different, although Diets I–IV fed to adults consistently produced bands 4, 10, and 11. The unique band of Diet I was band 1, and the predominant bands were bands 3, 9, and 11. The unique bands of Diet II were bands 7 and 8, and the predominant bands were bands 8 and 11. The predominant bands of Diet III were bands 3, 4, and 11. The unique band of Diet IV was band 12, and the predominant bands were bands 4, 6, and 11. The shared bands for the two larval diets were bands 13, 14, 30, 32, 36, and 37. The unique bands of Diet V were bands 16, 17, 19, 22, 29, 31, and 34, and the predominant bands were bands 13, 14, 19, 22, 30, and 32. The unique bands of Diet VI were bands 15, 18, 20, 21, 23, 26, 33, and 35, and the predominant bands were bands 13, 20, 24, 30, and 37.

**Table 3.**
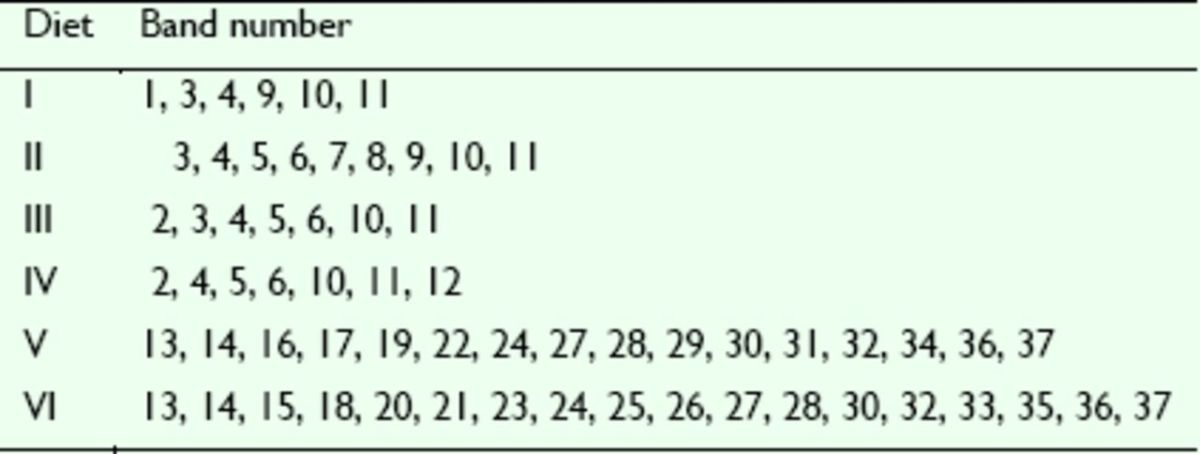
DGGE bands detected in gut samples of the larvae and adults of *D. helophoroides* fed different diets

Diets I–IV were the adult diets, Diets V and VI were the larval diets.

### Sequence analysis of DGGE bands

In this study, 25 bands were isolated from the DGGE profiles of *D. helophoroides* larvae (the band 23 signal was weak, with sequencing failure), and 12 bands were isolated from those of the adults (the band 10 and 12 signals were weak, with sequencing failure). The results of the sequencing are shown in Table 4 . After analysis of the sequences, the intestinal bacteria of the larvae were classified into four groups ( Table 4 ): Firmicutes (bands 17, 18, 20, 21, 24, 26, 28, 31, 32, 36, and 37), Proteobacteria (bands 15, 22, 29, 34, and 35), Fusobacteria (band 33), and unculturable bacteria (bands 13, 14, 16, 19, 25, 27, and 30). The adult intestinal bacteria were classified into two groups: Firmicutes (bands 1, 2, 3, 4, 5, and 7) and Proteobacteria (bands 6, 8, 9, and 11).

**Table 4.**
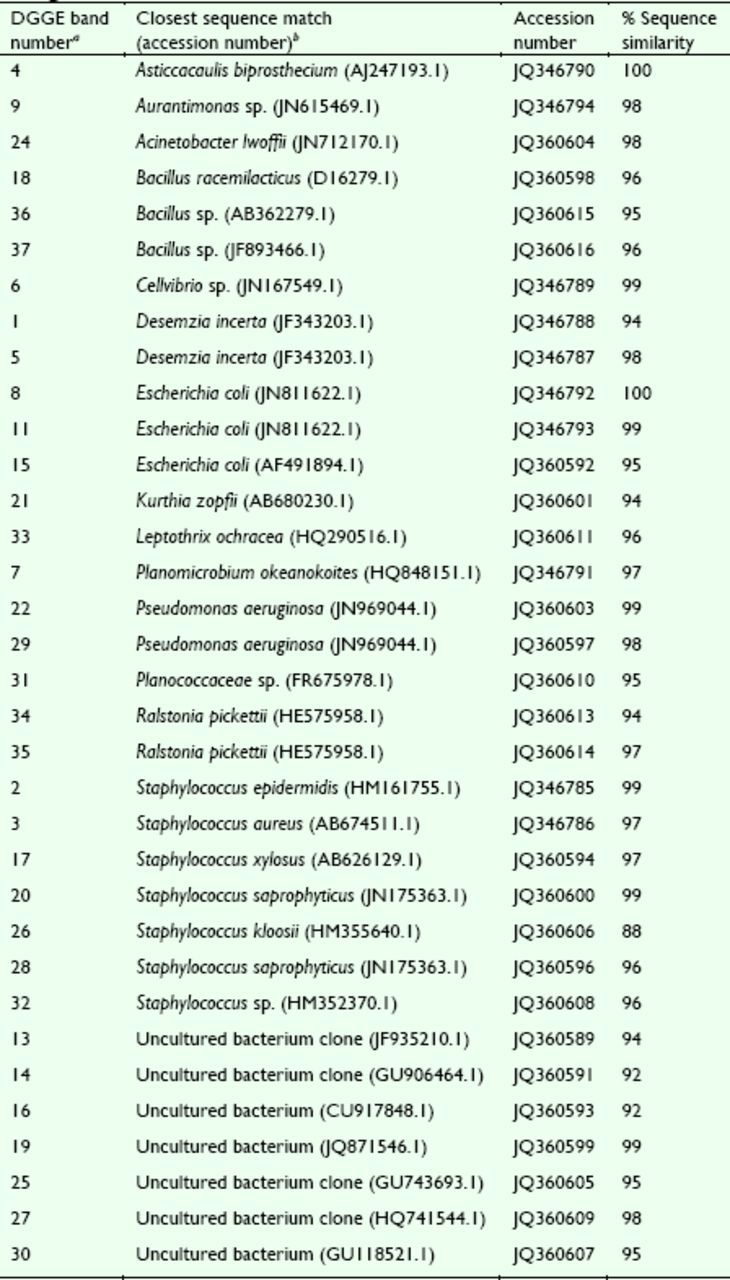
Results of the sequence analysis of the DGGE bands labeled in Fig. 3 .

*a* Bands 1–12 were obtained from gut samples of adults fed Diets I–IV, bands 13–37 were obtained from gut samples of larvae fed Diets V and VI; see Table 3 .

*b* Closest sequence match determined by NCBI nucleotide blast search

### Similarity analysis and biodiversity of bacterial communities

Fig. 3 was digitized and analyzed using Quantity One software (version 4.6.2, Bio-Rad). The similarity of the intestinal bacterial communities among the adults fed different diets are shown in Table 5 . The number of DGGE bands was taken as an indication of the number of species in each sample. The relative surface intensity of each DGGE band and the sum of all the surfaces for all bands in a sample were used to estimate species abundance ( Fromin et al. 2002 , Sekiguchi et al. 2002 ). DGGE profiles of the guts are shown in Fig. 3 . Many DGGE bands were observed in the profiles, thus indicating the presence of different bacterial populations and different relative abundance of species in the guts. As indicated by the values of Shannon indices, different diets appeared to affect the genetic diversity of the bacterial communities. Of the four diets fed to the adults, that with ant powder (Diet IV) and that with silkworm pupa powder (Diet I) as the major component pro duced the highest ( *H* Diet IV = 1.905) and the poorest ( *H* Diet I = 1.634) biodiversity, respectively. Cricket powder (Diet II) and darkling beetle pupa powder (Diet III) as the major component produced intermediate Shannon indices of 1.799 and 1.824, respectively. Comparison of the two diets fed to the larvae showed that the diversity of intestinal bacteria in the larvae fed the darkling beetle pupae ( *H* Diet V = 2.597) was less than the diversity in the larvae fed the artificial diet ( *H* Diet VI = 2.731).

**Table 5.**
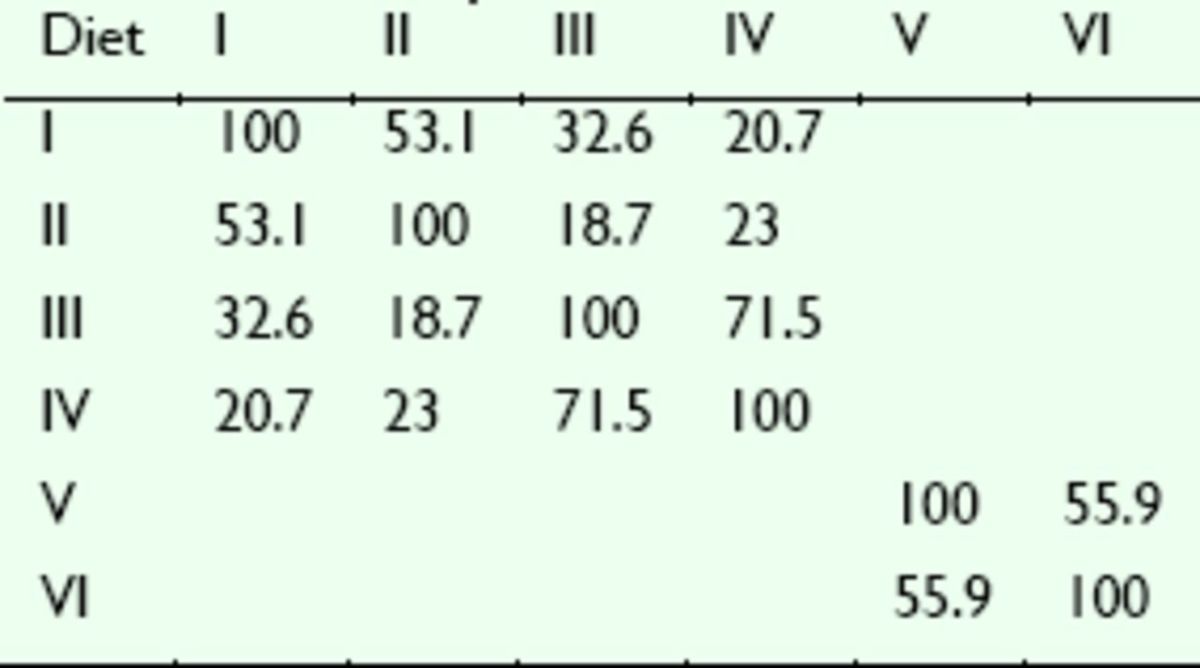
Similarity *(%)* matrix of the intestinal bacteria of *D. helophoroides* fed different diets Diet

Diets I–IV were the adults diets, Diets V and VI were the larval diets.

## Discussion

DGGE was used to reveal the structure of the intestinal bacteria community of *D. helophoroides* . The number of bands present can illustrate the diversity of the bacterial community, and the strength of the bands reflects the abundance of bacteria present, which can be used to determine the species, number of individuals, and microbial diversity ( Xing et al. 2006 ). The adult diets contained complex molecules, including cellulose, sugar, hemicellulose, pectin, and polysaccharides, and a wide diversity of microflora is required to ferment these large molecules. In this study, seven types of bacteria were isolated from the guts of adult *D. helophoroides,* including *Escherichia, Desemzia, taphylococcus, Asticcacaulis, Cellvibrio, Aurantimonas,* and *Planomicrobium. Escherichia* and *Staphylococcus* were the predominant bacterial communities in the adults in all of the treatments. *Aurantimonas* was found only in the guts of the adults that were fed Diets I and II. *Asticcacaulis* was present only in the guts of the adults that were fed Diets III and IV. *Staphylococcus aureus* existed in the guts of the adults that were fed Diets I, II, and III. *Cellvibrio* was not found in the guts of adults that were fed Diet I. Silkworm pupa powder and sunflower seed oil were the essential components of Diet I ( Table 1 ), and the predominant bacterial communities were *Escherichia, Desemzia, Staphylococcus, Asticcacaulis,* and *Aurantimonas* . Powdered crickets were the essential component of Diet II, and *Escherichia, Desemzia, Staphylococcus, Cellvibrio, Aurantimonas,* and *Planomicrobium* were the predominant bacterial communities. In Diet III, the main component was darkling beetle pupa powder, and the predominant bacterial communities were *Escherichia, Desemzia, Staphylococcus, Asticcacaulis,* and *Cellvibrio* . In Diet IV, ant powder was the essential component, and the predominant bacterial communities were *Escherichia, Desemzia, Staphylococcus, Asticcacaulis,* and *Cellvibrio.*

Twenty-four types of bacteria were isolated from the guts of larvae of *D. helophoroides,* belonging to *Escherichia, Bacillus, Staphylococcus, Kurthia, Planococcaceae, Ralstonia, Leptothrix, Acinetobacter,* and *Pseudomonas.* Larval Diet V contained darkling beetle pupae, and the predominant bacterial communities were *Bacillus, Staphylococcus, Planococcaceae, Ralstonia, Acinetobacter,* and *Pseudomonas* . The ingredients of Diet VI are listed in Table 2 , and the predominant bacterial communities were *Escherichia, Bacillus, Staphylococcus, Kurthia, Ralstonia, Leptothrix,* and *Acinetobacter.* Thus, the intestinal microflora changes when the diet changes. Previous studies on the microbial communities in the guts of crickets ( Domingo et al. 1988a , b; Kaufman et al. 2000 ), gypsy moths ( Broderick et al. 2004 ), cockroaches ( Kane and Breznak 1991 ), and wasps ( Reeson et al. 2003 ) showed that these microbes are essentially extrinsic and, as such, their composition transitorily varies with the surroundings and diet. A similar conclusion could be reached for the larval and adult midgut of *D. helophoroides.*

In the present work, many sequences showed > 98% homology to those of the phylotypes in GenBank; however, a significant proportion of the *D. helophoroides* intestinal bacterial sequences showed only 88-97% homology identity with the bacterial *16S rDNA* sequences in GenBank or could not be assigned at all. These sequences might be newly reported ( Broderick et al. 2004 ). Given that the sequences cover the variable V3 region of the bacterial *16S rDNA* gene, which has been shown to be a good indicator of phylogeny ( Mincer et al. 2004 ), there is still the possibility that some phylotypes may be unique to the guts of *D. helophoroides,* though unculturable forms may exist. Molecular techniques, such as PCR-DGGE, have revealed a diversity of unculturable bacterial species in a variety of insects, for example, honeybees ( Jeyaprakash et al. 2003 ), termites ( Hongoh et al. 2003 ), crickets ( Domingo et al. 1988a , b), and wood wasps ( Reeson et al. 2003 ). The signals for bands 10, 12, and 23 of the DGGE profiles were weak, resulting in sequencing failure; this situation might be caused by the amount of bacteria and intrinsic defects of the molecular techniques ( Riemann et al. 1999 ). In general, it is widely accepted that identical electromorphs represent identical gene sequences in DGGE profiles ( Dillon et al. 2010 , Arcuri et al. 2013 ); however, because the limitation of DGGE and intrinsic defects of the molecular techniques are obvious, it is also possible that one band represents more than one genotype, as PCR products of very different gene sequences may denature at the same point ( Reeson et al. 2003 ). In addition, DGGE bands with short sequences (about 150 bp in the present study) may have affected the resolution of the taxa and limited the performance of the analysis ( Asakawa and Kimura 2008 ).

Most of the bacteria isolated in this study have also been found in other insects. Species of the genus *Staphylococcus* , which are Gram-positive bacteria, often can be found in insect guts, and most of them are not pathogenic. Eutick et al. (1978) examined the intestinal bacteria of 10 species of Australian termites and found that *Staphylococcus* was the predominant bacterium. *Staphylococcus* was also isolated from the gut of *A. germari* by using traditional culture-dependent techniques ( Zhang et al. 2004 ) and from *Hepialus gonggaensis* Fu & Huang by using culture-dependent and culture-independent techniques ( Zhuo et al. 2004 ). Therefore, *Staphylococcus* may be a normal intestinal bacterium of many insects. He et al. (2001) and Zhang et al. (2004) found *Staphylococcus, Pseudomonas,* and *Escherichia* in the gut of *A. germari.* Broderick et al. (2004) found *Staphylococcus, Pseudomonas,* and *Escherichia* in the gut of *Lymantria dispar* (L.), and Dillon et al. (2008) found *Acinetobacter* in acridids. Other genera have been little researched in the guts of insects to date, yet it is possible that the herein detected phylotypes are unique to *D. helophoroides* guts. The observed differences between the intestinal bacterial communities of larvae and adults of *D. helophoroides* , the presence of unique bacterial phylotypes, and the influence of diet on the composition of the intestinal microflora merit further investigation.
